# BioFire FilmArray BCID2 versus VITEK-2 System in Determining Microbial Etiology and Antibiotic-Resistant Genes of Pathogens Recovered from Central Line-Associated Bloodstream Infections

**DOI:** 10.3390/biology11111573

**Published:** 2022-10-26

**Authors:** Heba M. El Sherif, Mahitab Elsayed, Mona R. El-Ansary, Khaled M. Aboshanab, Mervat I. El Borhamy, Khaled M. Elsayed

**Affiliations:** 1 Department of Microbiology, Faculty of Pharmacy, Misr International University (MIU), Cairo 19648, Egypt; 2 Department of Clinical Pharmacy, Faculty of Pharmacy, Modern University for Technology and Information (MTI), Cairo 12055, Egypt; 3 Department of Biochemistry, Modern University for Technology and Information (MTI), Cairo 12055, Egypt; 4 Department of Microbiology and Immunology, Faculty of Pharmacy, Ain Shams University, Organization of African Unity Street, Cairo 11566, Egypt; 5 International Medical Center, Clinical Microbiology Laboratory, Cairo 19648, Egypt

**Keywords:** CLABSI, FilmArray BCID2, VITEK-2 system, identification, resistance genes

## Abstract

**Simple Summary:**

Central line-associated bloodstream infection (CLABSI) is among the most serious hospital-acquired infections that impose public health threats. Accordingly, the urgent need for a rapid identification method that can identify CLABSI pathogens and their resistance genetic markers is essential for the prompt initiation of adequate antibiotic therapy. This study aimed to evaluate the clinical performance of the BioFire FilmArray Blood Culture Identification 2 (BCID2) panel in the rapid identification of 33 microbial species and 10 antibiotic resistance genes in comparison to the VITEK-2 system, among 104 patients admitted to an ICU in an Egyptian tertiary care hospital with bloodstream infections (BSIs) within 48 h of a central line placement. In comparison to the VITEK-2 system, the BCID2 panel showed an overall sensitivity of 75.8% and an overall specificity of 98% in detecting microbial species. The sensitivity and specificity for detecting resistance genes by BCID2 were 90% and 99.6%, respectively. In conclusion, this study emphasizes the high sensitivity and specificity of the BCID2 as compared to the VITEK-2 system in the rapid and reliable microbial identification, as well as the accurate detection of various antibiotic resistance markers.

**Abstract:**

Central line-associated bloodstream infection (CLABSI) is among the most serious hospital acquired infections. Therefore, the rapid detection of the causative microorganism is of crucial importance to allow for the appropriate antimicrobial therapy. In the present study, we analyzed the clinical performance of the BioFire FilmArray Blood Culture Identification 2 (BCID2) panel in the identification of 33 microbial species and 10 antibiotic resistance genes in comparison to the VITEK-2 system. A total of 104 blood specimens were included. The FilmArray BCID2 results were concordant with the VITEK-2 system in 69/97 specimens (71.1%). Non-concordance was either due to the detection of more pathogens by the FilmArray BCID2 23/28 (82%) or microbial species were misidentified 5/28 (18%). Hence, in comparison to the VITEK-2 system, the FilmArray BCID2 panel showed an overall sensitivity of 75.8% (95% CI, 66–83%) and an overall specificity of 98% (95% CI, 97–98.8%) in detecting microbial species. For the resistance genes, the FilmArray BCID was able to detect the presence of *bla*CTX-M gene in 23 Gram-negative isolates, *bla*NDM and *bla*OXA-48- like genes in 14 and 13 isolates, respectively. The *mec*A and *mec*C genes were found in 23 *Staphylococcus* species, while *mec*A, *mec*C and MREJ genes were found in 4 *Staphylococcus aureus* isolates. The sensitivity and specificity for detecting resistance genes by the FilmArray BCID2 was 90% (95% CI, 81.4–95%) and 99.6% (95% CI, 99–100%), respectively. As concluded, the present study emphasizes the high sensitivity and specificity of the FilmArray BCID2 in the rapid and reliable detection of different bacteria and fungi from positive blood culture bottles, as well as the accurate detection of various antibiotic resistance markers.

## 1. Introduction

Bloodstream infections (BSIs) present a major public threat worldwide. These devastating infections are one of the leading causes of morbidity and mortality in patients of all ages, particularly in critically ill and immunocompromised patients [1]. Central venous lines (CVLs) are commonly used among hospitalized patients, especially patients in intensive care units (ICUs) [1]. They have a high risk of adverse events with infectious complications ranging from localized skin infections to developing central line-associated bloodstream infection (CLABSI), which is one of the most frequent and fatal healthcare-associated infections (HAIs) [2,3]. CLABSIs are developed in patients with CVLs with no other source of bacteremia. Due to their relatively high incidence and high mortality rate in Egypt, it becomes very crucial to reduce the rate of these infections in ICU settings [4,5].

Globally, the key pathogens for BSIs are Staphylococcus aureus (*S. aureus*), Escherichia coli (*E. coli*), Klebsiella pneumoniae (*K. pneumoniae*), Pseudomonas aeruginosa (*P. aeruginosa*), and coagulase-negative staphylococci [5]. Several studies have reported the highest incidence for *E. coli* especially as regards hospital-acquired infections, while *S. aureus* and P. aeruginosa are among the prominent causes of mortality [6]. Major dramatic changes have emerged with *E. coli* and *K. pneumoniae* epidemiology in BSIs owing to their resistance to third-generation cephalosporins, which is primarily due to the production of the extended-spectrum beta-lactamase (ESBL) enzymes. In 2019, the Center for Disease Control and Prevention (CDC) selected ESBL-producing Enterobacteriaceae to be a “serious threat” pathogen [7,8]. Thus, the high prevalence of infections caused by ESBL-producing Enterobacteriaceae lead to the extensive use of carbapenems in clinical practice. Consequently, the resistance of Gram-negative pathogens to carbapenems has widely developed and the emergence of carbapenemase-resistant Enterobacteriaceae (CRE) became a more serious problem [9]. Furthermore, the widespread infections caused by methicillin-resistant *S. aureus* (MRSA) and the massive use of vancomycin as one of the important first-line treating options forced not only the emergence of vancomycin-intermediate *S. aureus* (VISA), but also for the substantial rise of complete resistance to vancomycin in recent years. Therefore, preventing the dangerous spread of these dreadful superbugs became a major challenge facing all healthcare workers worldwide [10].

The rapid and accurate detection of the causative agent from a positive blood culture can expedite appropriate antimicrobial treatment, improve patient outcomes, decrease hospitalization and healthcare costs, and reduce the risk of the development of antimicrobial resistance [11]. The use of blood cultures remains the gold standard to identify the causative pathogens in BSIs [12]. However, traditional blood cultures and conventional antibiotic susceptibility testing are time-consuming methods as they require multiple incubation steps [13]. Alternatively, automation in blood culture systems that monitors the growth monitoring of the organisms is needed to detect positive blood cultures [14].

The VITEK-2 system (bioMérieux. Marcy l’Etoile, France) used in the present study is an automated system for identification and antimicrobial susceptibility testing after a standardized inoculum has been loaded into the system. The results are available within 3 h for identification and 18 h for susceptibility testing [15]. However, the crucial need for speeding up the process of identification and antimicrobial susceptibility of the positive blood cultures lead to more recent advances that allow more rapid identification within 1 h. The BioFire FilmArray blood culture ID (BCID2; bioMérieuxm Marcy l’Etoile, France) is a highly multiplexed PCR kit that identifies 33 species, including 28 bacterial species and five Candida species. Furthermore, the BioFire FilmArray BCID2 can detect 10 genetic resistance markers including methicillin resistance genes in *Staphylococci* and vancomycin resistance genes in enterococci (*mec*A/*mec*C (a gene A or C that produces a mutated pencillin binding protein coded for methicillin resistance), *mec*A/*mec*C & MREJ (the gene coded by *mec* right-extremity junction containing the right-extremity of SCCmec and orfX, chromosomal *S. aureus* gene, and *van*A), carbapenemase-resistant genes including those coded for, *K. pneumoniae* carbapenemase (*bla*KPC), imipenem-resistant *Pseudomonas*-type carbapenemase (*bla*IMP), New Delhi metallo-β-lactamase (*bla*NDM), oxacillinase type crabapenemase *(bla*OXA-48-like) Verona integron-encoded metallo-β-lactamase (*bla*VIM) in Enterobacterales, *P. aeruginosa* and *Acinetobacter baumannii* (*A. baumannii)*, the most common ESBL gene (*bla*CTX-M), and a genetic marker for colistin resistance (*mcr*-1) [11,16]. In this study, we evaluated the clinical performance of the BioFire FilmArray Blood Culture Identification 2 (BCID2) panel in comparison with the VITEK-2 system with respect to pathogen identification and the presence of antibiotic resistance genes among bacteremic patients having CLABSIs.

## 2. Materials and Methods

### 2.1. Study Design and Inclusion Criteria

This study was conducted at the International Medical Center (IMC, Cairo, Egypt), a tertiary care hospital with 800 beds and 10 different intensive care units (ICUs). The study period included a total of six consecutive months between January 2021 and July 2021. This study was conducted in accordance with the ethical principles stated in the Declaration of Helsinki and was approved by the institutional ethical committee, Faculty of Pharmacy, Ain Shams University (ENREC-ASU-2018-72).

A total of 104 blood culture specimens were collected (BacT/ALERT FAN Plus aerobic or anaerobic bottles, bioMérieux, Marcy l’Etoile, France) from adult patients admitted to ICUs. Inclusion criteria included all blood cultures from patients over the age of 18 years and with BSIs not related to an infection at another site that develops within 48 h of a central line placement [17]. Blood cultures were taken during an episode of suspected bacteremia; only the bottles which flagged positively were included in the study.

### 2.2. Microbiological Procedures

Standard operating procedures were followed for the processing of conventional cultures. Accordingly, all positive blood culture bottles were subjected to Gram staining. The positive cultures were subcultured in Trypticase soy agar supplemented with 5% sheep blood, chocolate agar, and MacConkey agar (Oxoid, UK). If yeasts were detected on the Gram stain, inoculation on the Sabouraud agar plate (Oxoid, UK) was also performed. Plates were incubated at 37 °C for a maximum incubation period 48 h. Furthermore, anaerobic blood culture bottles were inoculated onto 10% sheep blood and chocolate agar plates and incubated at 37 °C in 5% CO2 [12,18].

The identification of isolates was performed using conventional biochemical tests such as catalase, coagulase, and DNase to differentiate between Gram-positive bacteria. For Gram-negative bacteria, sugar fermentation, indole production, triple sugar iron agar, as well as an oxidase test, were performed to differentiate between different Gram-negative bacteria. Identification was also performed using the VITEK-2 instrument (bioMérieux. Marcy l’Etoile, France) according to the manufacturer’s instructions. Briefly, pure isolated colonies were picked up from the solid culture media. The turbidity of the bacterial suspension was adjusted with a VITEK DensiCHEK™ colorimeter (bioMérieux, Marcy l’Etoile, France) to match the McFarland 0.5 standard in 0.45% sodium chloride. The bacterial suspensions were inoculated into the following specific identification cards of the automated VITEK-2 system using the standard protocol: Gram-positive cocci (GPC), Gram-positive bacilli (GPB), Gram-negative bacilli (GNC), and yeasts (YST). The VITEK-2 system reported the results automatically with the software release 2.01 [19,20].

For the susceptibility testing, 2 mL samples of each suspension were prepared as described above and were automatically loaded into the VITEK-2 AST system (bioMérieuxm Marcy l’Etoile, France) using the GN04 and P526 cards for the susceptibility testing of GNB and for GPC, respectively, and the 2.01 release software. The cards were read by kinetic fluorescence measurement and the results were reported after overnight incubation [20]. Antimicrobial susceptibility data were interpreted according to the CLSI guidelines, 2020 breakpoints [21].

### 2.3. BioFire FilmArray BCID2 Testing

The FilmArray BCID2 testing was performed according to the manufacturer’s guidelines. This involved loading hydration solution into the pouch, followed by 200 μL of broth from a positive blood culture bottle. Then, the provided sample buffer was added to the sample injection well. The BCID2 pouch was then loaded into the BioFire FilmArray instrument (Release Version 2, Software Module Version: BioFire FilmArray FA Link UI 2.1.273.0). Nucleic acid extraction, multiplex PCR, and an analysis of DNA melting curves to confirm and identify the presence of bacterial and fungal isolates, as well as antimicrobial resistance genes, were all performed by the instrument within 2 h [16].

### 2.4. Statistical Data Analysis

The correlation between BCID2 and VITEK-2 was investigated in the form of a positive percent agreement (PPA) and a negative percent agreement (NPA) at 95% confidence intervals (95% CI), by modified Wald method in GraphPad Prism^®^ version 5.00. The PPA = [true positive/(true positive + false negative)] × 100% and NPA = [true negative/(true negative + false positive)] × 100% [18].

## 3. Results

### 3.1. Study Population

A total of 104 blood specimens were included in this study. All blood specimens were received from patients admitted to four adult ICUs (medical ICU, surgical ICU, cardiothoracic ICU, and neurosurgery ICU). Most of the specimens were obtained from male patients (n = 65, 62.5%). The mean age of patients included in the study was 61 years (range was from 18 to 99 years).

### 3.2. Identification of Microbial Isolates

Of the 104 blood specimens, six blood culture bottles failed to have any evidence of microbial growth and one blood culture bottle revealed the presence of *Candida parapsilosis* and *Candida auris* with BCID2 only, but they were not detected with the VITEK-2. Out of the 97 positive blood specimens, 94 were monomicrobial, with the predominance of Gram-positive cocci (GPC) (40/94, 42.6%), followed by Gram-negative bacilli (GNB) (45/94, 47.9%), and 9/94 (9.6%) isolates were identified as different *Candida* species, which were identified at the species level in both the VITEK-2 and the BCID2, as shown in Table 1. Three blood specimens had polymicrobial isolates. The most identified bacterial species from the monomicrobial isolates in the BCID2 panel were *K. pneumoniae* (n = 19), followed by *S. epidermidis* (n = 16) and *E. coli* (n = 14). These results were in accordance with the VITEK-2 results.

### 3.3. Discordant Identification

Of the 97 blood cultures identified by the VITEK-2, the BCID2 showed concordant results in 69 cases (71.1%) and 28 were discordant (28.9%). Non-concordance was either due to the detection of additional pathogens 82% (23/28) by the FilmArray BCID2, or microbial species were misidentified 18% (5/28). As shown in Table 2, all GNB monomicrobial isolates identified by the VITEK-2 could be identified in the BCID2 panel, except for one *Salmonella* spp. that could be identified by the BCID2 alone (study no. 1). Additionally, in the 15 of the GNB monomicrobial cases, the BCID2 identified additional isolates that were not detected by the VITEK-2 system. Two of the GPC isolates were identified as *Staphylococcus aureus*, and *S. epidermidis* in the VITEK-2 system, but were identified as *S. aureus* and *Enterococcus faecalis*, and *Staphylococcus*. spp. and *Candida albicans*, respectively, in the BCID2 panel (study no. 40 and 74). Discrepancies resulted from the identification of coagulase-negative Staphylococci (CoNS) but no concordant species identification (i.e., the BCID2 identified *S. epidermidis* while the VITEK-2 identified a different CoNS species; n = 4) (studies no. 8, 13, 45, and 67). Another discrepant result was identified as *A. baumannii* in the VITEK-2 system, whereas the same isolate was identified as *Enterobacter* spp. in the BCID2 panel (study no. 83).

Despite the discrepancies in *Candida* identification, both the VITEK-2 and the BCID2 were able to identify different *Candida* spp. except for study no. 74, where the VITEK-2 identified *S. epidermidis*, while the BCID2 panel identified *Candida albicans* as well as *Staphylococcus* spp. Moreover, in study no. 60, no growth was detected with the VITEK-2, while the BCID2 detected *Candida auris*, and *Candida parapsilosis*.

Three blood specimens had polymicrobial isolates. Of these three microbial isolates, one discordant result was obtained between the VITEK-2 and the BCID2 panels. In this case, the BCID2 panel identified three extra discordant isolates (*Staphylococcus* spp., *Enterococcus faecium*, and *Candida glabrata*), in addition to *K. pneumoniae* and *Escherichia coli*, whereas the VITEK-2 system identified only *K. pneumoniae* and *Escherichia coli* (study no. 54) (Table 2). Discrepancies were also observed in the case of the two other polymicrobial specimens as the VITEK-2 could identify CoNS and *Enterococcus* to the species level, whereas the BCID2 panel only identified them as *Staphylococcus* spp. and *Enterococcus* spp. The performance of the BCID2 was analyzed for mono and polymicrobial isolates, as shown in Table 3. The BCID2 showed an overall sensitivity of 75.8% and an overall specificity of 98% in comparison to the VITEK-2.

### 3.4. Detection of Resistance Genes

The BCID2 panel can detect 10 various genetic markers associated with acquired resistance phenotypes (*mec*A/C, *mec*A/C & MREJ, *van*A, *bla*KPC, *bla*IMP, *bla*NDM, *bla*OXA-48-like and *bla*VIM, *bla*CTX-M and *mcr*-1). As shown in Table 4, the BCID2 was able to detect 23 Gram-negative third-generation cephalosporin-resistant isolates harboring *bla*CTX-M gene: *E. coli* (n = 9) and *K. pneumoniae* (n = 14). The BCID2 was also able to detect 26 carbapenem-resistant isolates: *E. coli* (n = 3); one isolate harboring both *bla*OXA-48-like and *bla*NDM, one isolate harboring *bla*OXA-48-like only, and one isolate harboring *bla*NDM only, *K. pneumoniae* (n = 22); eight isolates harboring both *bla*OXA-488 and *bla*NDM, three isolates harboring *bla*OXA-48 only, and three isolates harboring *bla*NDM only, *A. baumannii* (n = 1). The BCID2 could also detect 27 *Staphylococcus* species harboring *mec*A/C or *mec*A/C & MRJE: *S. aureus* (n = 4) harboring *mec*A/C and MREJ and other *Staphylococcus* spp. (n = 23) harboring *mec*A/C genes.

### 3.5. Discordant Genotypic Results Obtained by VITEK-2 System and BCID2 Panel

The discordant genotypic results obtained by the VITEK-2 system and the BCID2 panel VITEK-2 system could identify six methicillin-resistant *Staphylococcus* spp. (cefoxitin-resistant) that were found to be *mec*A/C negative by the BCID2 panel. Moreover, the BCID2 failed to detect the *bla*CTX-M gene in 1 *K. pneumoniae* and three *E. coli* that were found to be ESBL positive by the VITEK-2 system (Table 5). Table 6 compares the accuracy of the BCID2 panel to that of the VITEK-2 in terms of sensitivity and specificity, where the total PPA and NPA were 90% and 99.6%, respectively.

## 4. Discussion

Central line-associated bloodstream infection (CLABSI) is a highly prevalent problem in ICUs. It presents a high burden on the healthcare system as it is a major reason behind prolonged patient hospitalization, increased financial burden, as well as a higher risk of mortality [22]. The urgent need for a rapid identification method that can identify CLABSI pathogens and their resistance genetic markers, among ICU patients, is essential for the prompt initiation of adequate antibiotic therapy. This can improve patient outcomes, reduce mortality rates and prolonged hospital stays, and limit broad-spectrum antibiotic usage that represents a high economic burden [11]. We designed this comparative study to evaluate the performance of the BCID2 in the rapid identification of bloodstream pathogens and their most common resistance genetic markers. Our BCID2 has been shown to rapidly identify pathogens and relevant antimicrobial resistance genetic markers within only 2 h. In contrast, the VITEK-2 requires an additional 24 h for identification and antimicrobial susceptibility testing. The microbial profile of our study revealed the predominance of GNB (47.9%) over GPC (42.5%) causing CLABSIs as detected using the VITEK-2. Among the recovered Gram-negative isolates, the highest prevalence was for *K. pneumoniae* (42.2%), followed by *E. coli* (28.8%). Our results were in accordance with those observed by Al-khawaga et al. 2021, and Venturini et al. 2016, as members of *Enterobacteriaceae* were the most common causes of CLABSI [5,23]. Many of the previously published studies worldwide reported the changing epidemiology of BSIs towards Gram-negative organisms, which could be attributed to the prevalence of nosocomial infections caused by the multidrug-resistant *Enterobacteriaceae* among patients admitted to the ICU in tertiary care hospitals [5,24]. However, in our study, the BCID2 was able to detect higher frequencies of some of the GNBs, such as *A. baumannii*, *E. coli* and *P. aeruginosa*, and *Enterobacter cloacae*, and was able to detect one *Salmonella* spp. that was not detected by the VITEK-2. Our results revealed an overall concordance in species identification between the BCID2 and the VITEK-2 system of 71.1% particularly, in monomicrobial specimens. In 2021, Berinson et al. reported that the concordance in identification between the BCID2 and MALDI-TOF mass spectrometry was 94% [11]. In our study, the performance of the BCID2 in microbial identification compared to the VITEK-2 system was analyzed. Our data revealed that the overall sensitivity of the BCID2 panel was 75.8% with an overall specificity of 98% in comparison to the VITEK-2. Our results were in agreement with Holma et al., where the sensitivity and specificity of the BCID2 panel were 98.8% and 99.9%, respectively [25]. In another study, Altun et al. reported the sensitivity of the FilmArray for Gram-positive and Gram-negative bacteria was 96.7% and 98.5%, respectively, while the specificity was 93.7% and 100%, respectively [26].

Recently, coagulase-negative staphylococci have been recognized as a true pathogen in multiple sites of infection rather than being dismissed as a contaminant [27]. In our study, the results of the BCID2 panel revealed a high prevalence of coagulase-negative Staphylococci where 18 *Staphylococcus* spp. and 16 *S. epidermidis* were detected. Several studies focused on the high prevalence of coagulase-negative staphylococci as one of the frequent causes of CLABSI. In 2021, Rule et al. reported that coagulase-negative staphylococci were the most frequent organisms recovered from blood specimens [16]. Similarly, Kendirli et al. 2017, and Dao et al. 2018, found that coagulase-negative staphylococci were the most common cause of CLABSI among children [28,29]. However, not all coagulase-negative staphylococci could be considered as the causes of clinically significant bacteremia. The benefit of the BCID2 is in detecting whether the coagulase-negative staphylococci is a contaminant or a true pathogen by detecting the *mec*A/*mec*C genes. Thus, allowing the rapid initiation of antimicrobial therapy with vancomycin or the earlier cessation of unwanted antimicrobial therapy is a crucial approach to limiting antimicrobial resistance [16]. Compared to the results of the VITEK-2 system, discordant BCID2 results were related to misidentified or additionally identified microbial species. Among the coagulase-negative Staphylococci, the BCID2 panel only identifies *S. epidermidis*, while the rest of the coagulase-negative staphylococci were identified as *Staphylococcus* spp. Our results were in accordance with Kang et al., 2020, where two *S. epidermidis*, two *S. hominis*, one *S. caprae*, and one *S. lugdunensis*, were identified as *Staphylococcus* spp. [12]. Moreover, in 15 of the GNB monomicrobial cases, the BCID2 identified additional isolates that were not detected by the VITEK-2 system. This reveals one of the major strengths of the BCID2 panel in identifying multiple pathogens simultaneously from polymicrobial blood culture bottles [26]. When analyzing the performance of the BCID2 in detecting the five resistance genetic markers that were found among our isolates, the results revealed an overall sensitivity of 90% and an overall specificity of 99.6%, which indicates the ability of the BCID2 to correctly detect and identify these specific genes that are frequently encountered in BSI isolates. Thus, this reveals a potential strength for the BCID2 in detecting the underlying cephalosporin and carbapenem resistance in Gram-negative pathogens, as well as methicillin and vancomycin resistance among *Staphylococcus* spp. Similarly, Holma et al. reported an excellent performance of the BCID2 panel with an overall 100% sensitivity and specificity for antimicrobial resistance genes [25]. However, there are a few limitations for the BCID2, such as the inability of the BCID2 to identify some CoNS to the species level. Moreover, it failed in the detection of the *mec*A/*mec*C genes in six *Staphylococcus* spp. that were found to be cefoxitin resistant by the VITEK2 system. Additionally, the *bla*CTX-M gene was not detected in four Gram-negative pathogens that were found to be ESBL-positive by the VITEK2 system.

## 5. Conclusions

The present study highlights the high sensitivity and specificity of the FilmArray BCID2 in the rapid and reliable detection of bacteria and yeast from positive blood culture bottles, as well as the accurate detection of various antibiotic resistance markers. Accordingly, the FilmArray BCID2 proved to be a valuable tool that may aid in optimizing antimicrobial therapy, reducing antibiotic resistance as well as improving patient outcomes.

## Figures and Tables

**Table 1:** Identification of specimens with monomicrobial isolates (N = 94).

Gram-Stain	VITEK-2	N	BCID2	N
GPC	*S. aureus*	4	*S. aureus*	5
	*S. hominis*	6	*Staphylococcus* spp.	18
	*S. saprophyticus*	6	*-*	-
	*S. hemolyticus*	3	*-*	-
	*S. epidermidis*	16	*S. epidermidis*	16
	*Streptococcus pneumoniae*	1	*Streptococcus pneumoniae*	0
	*Streptococcus agalactia*	1	*Streptococcus* spp.	4
	*Enterococcus faecium*	1	*Enterococcus faecium*	2
	*Enterococcus faecalis*	2	*Enterococcus faecalis*	6
	*Enterococcus* spp.	0	*Enterococcus* spp.	2
Total GPC		40		53
GNB	*K. pneumoniae*	19	*K. pneumoniae*	19
	*A. baumannii*	5	*A. baumannii*	8
	*E. coli*	13	*E. coli*	14
	*P. aeruginosa*	3	*P. aeruginosa*	4
	*Salmonella* spp.	0	*Salmonella* spp.	1
	*Serratia marcescens*	3	*Serratia marcescens*	3
	*Enterobacter cloacae*	2	*Enterobacter cloacae*	3
Total GNB		45		52
Yeast	*Candida parapsilosis*	5	*Candida parapsilosis*	6
	*Candida auris*	2	*Candida auris*	5
	*Candida glabrata*	1	*Candida glabrata*	2
	*Candida albicans*	0	*Candida albicans*	2
	*Candida troplicalis*	1	*Candida troplicalis*	1
Total yeast		9		16

BCID2, BioFire FilmArray Blood Culture Identification 2; N: number of isolates. GPC: Gram-positive cocci. GNB: Gram-negative bacilli.

**Table 2:** Overview on discordant species identification by VITEK-2 system and BCID2 panel.

Study No.	VITEK-2 Identification	BCID-2 Identification
1	*E. coli*	*K. pneumoniae*, *E. coli*, *Streptococcus pneumoniae*, *Salmonella* spp.
3	*K. pneumoniae*	*K. pneumoniae*, *A. baumannii*
8	*S. saprophyticus*	*S. epidermidis*
13	*S. hemoliticus*	*S. epidermidis*
24	*Pseudomonas aeruginosa*	*Pseudomonas aeruginosa*, *Enterococcus fecalis*
30	*K. pneumoniae*	*K. pneumoniae*, *A. baumannii*
31	*A. baumannii*	*A. baumannii*, *Staphylococcus* spp.
37	*E. coli*	*E. coli*, *Staphylococcus* spp.
40	*S. aureus*	*S. aureus*, *Enterococcus fecalis*
45	*S. hemoliticus*	*S. epidermidis*
49	*K. pneumoniae*	*K. pneumoniae*, *A. baumannii*, *Pseudomonas aeruginosa*
54	*K. pneumonia*, *E. coli*	*K. pneumoniae*, *E. coli*, *Staphylococcus* spp., *Enterococcus faecium*, *Candida glabrata*
55	*K. pneumoniae*	*K. pneumoniae*, *A. baumannii*
58	*K. pneumoniae*	*K. pneumoniae*, *Enterococcus faecalis*
60	None	*Candida auris*, *Candida parapsilosis*
61	*A. baumannii*	*A. baumannii*, *Staphylococcus* spp.
62	*Candida parapsilosis*	*Candida parapsilosis*, *Candida albicans*
64	*Candida parapsilosis*	*Candida parapsilosis*, *Candida tropicalis*
65	*K. pneumoniae*	*K. pneumoniae*, *E. coli*
67	*S. hominis*	*S. epidermidis*
68	*A. baumannii*	*A. baumannii*, *Candida auris*
74	*S. epidermidis*	*Staphylococcus* spp., *Candida albicans*
79	*Enterobacter cloacae*	*Enterobacter cloacae*, *Staphylococcus* spp.
81	*E. coli*	*S. aureus*, *E. coli*
83	*A. baumannii*	*Enterobacter cloacae*
99	*K. pneumoniae*	*K. pneumoniae*, *S. epidermidis*, *Enterococcus faecalis*
100	*K. pneumoniae*	*K. pneumoniae*, *Enterococcus faecalis*
102	*Candida parapsilosis*	*Candida parapsilosis*, *Candida auris*

BCID2, BioFire FilmArray Blood Culture Identification 2.

**Table 3:** Identification performance of each microorganism by BCID2 in comparison to VITEK-2.

Target Organism	NPA [95% CI]	PPA [95% CI]
*S. aureus*	99% (94–99)	100% (16.7–100)
*S. epidermidis*	92% (84–96)	68.7% (44–86)
*Enterococcus* spp.	94% (87–97)	100% (45–100)
*Candida* spp.	95.8% (89–98)	100% (62–100)
*S. hominis*	100% (95.5–100)	0 (0–4)
*S. saprophyticus*	100% (95–100)	0 (0–48)
*S. hemolyticus*	100% (95–100)	0 (0–48)
*K. pneumoniae*	98.9% (92.9–99)	95% (74.5–100)
*E. coli*	90% (82–94.6)	100% (74.8–100)
*Acinetobacter* spp.	95% (88.4–98)	80% (36–98)
*Pseudomonas* spp.	99% (94–100)	100% (38–100)
*Streptococcus* spp.	98% (92.7–100)	100% (29–100)
*Salmonella* spp.	100% (95.6–100)	100% (29–100)
*Serratia marscenes*	100% (95.6–100)	100% (38.2–100)
*Enterobacter* spp.	98% (92.7–100)	100% (29–100)
Total pathogens	98% (97–98.8)	75.8% (66–83)

PPA, positive percent agreement; NPA, negative percent agreement.

**Table 4:** Resistance marker detected by BCID2.

Isolate	Resistance Genes Detected by BioFire BCID2				
	*blaCTX-M*	*blaOXA-48*	*mecA/C*	*mecA/C& MREJ*	*blaNDM*
-Phenotypic 3rd generation Cephalosporin resistance					
*E. coli* (n = 9)	9	0	0	0	0
*K. pneumoniae* (n = 14)	14	0	0	0	0
-Carbapenam resistant isolates					
*E. coli* (n = 3)	0	2	0	0	2
*K. pneumoniae* (n = 22)	0	11	0	0	11
*A. baumannii* (n = 1)	0	0	0	0	1
-Methicillin resistant isolates					
*S. aureus* (n = 4)	0	0	0	4	0
*Staphylococcus* spp. (n = 23)	0	0	23	0	0

BCID2, BioFire FilmArray Blood Culture Identification 2; *blaCTX-M*, *extended spectrum beta-lactamase (cefotaximase*); *bla*NDM, New Delhi metallo-β-lactamase; *bla*OXA-48, oxacillinase type carbapenemase; *mec*A/*mec*C, a gene A or C that produces a mutated pencillin binding protein coded for methicillin resistance; MREJ, the protein coded by *mec* right-extremity junction (MREJ) (containing the right-extremity of SCCmec and orfX, chromosomal *S. aureus* gene); PPA, positive percent agreement; NPA, negative percent agreement.

**Table 5:** Discordant genotypic results obtained by the VITEK-2 system and BCID2 panel.

Study	VITEK-2		BCID2		
	Identification	Resistance Pattern	Identification	Concordant Identification	Resistance Gene Confirmation
5	*S. hominis*	Cefoxitin (+)	*Staphylococcus* spp.	Yes	*mecA/C* (−)
13	*S. haemolyticus*	Cefoxitin (+)	*S. epidermidis*	No	*mecA/C* (−)
18	*S. saprophyticus*	Cefoxitin (+)	*staphylococcus* spp.	Yes	*mecA/C* (−)
20	*S. Saprophyticus*	Cefoxitin (+)	*staphylococcus* spp.	Yes	*mecA/C* (−)
47	*S. haemolyticus*	Cefoxitin (+)	*staphylococcus* spp.	Yes	*mecA/C* (−)
50	*S. hominis*	Cefoxitin (+)	*staphylococcus* spp.	Yes	*mecA/C* (−)
7	*K. pneumoniae*	ESBL (+)	*K. pneumoniae*	Yes	*blaCTX-M* (−)
71	*E. coli*	ESBL (+)	*E. coli*	Yes	*blaCTX-M* (−)
81	*E. coli*	ESBL (+)	*E. coli*	Yes	*blaCTX-M* (−)
87	*E. coli*	ESBL (+)	*E. coli*	Yes	*blaCTX-M* (−)

BCID2, BioFire FilmArray Blood Culture Identification 2; *S. epidermidis*, Staphylococcus epidermidis; *S. hominis*, Staphylococcus hominis; *S. haemolyticus*, Staphylococcus haemolyticus; S. saprophyticus, Staphylococcus saprophyticus; *S. hominis*, Staphylococcus hominis; *K. pneumoniae*, Klebsiella pneumoniae; *E. coli*, Escherichia coli. ESBL, extended spectrum beta-lactamase; blaCTX-M, extended spectrum beta-lactamase (cefotaximase); blaNDM, New Delhi metallo-β-lactamase; blaOXA-48, oxacillinase type carbapenemase; mecA/mecC, a gene A or C that produces a mutated pencillin binding protein coded for methicillin resistance; MREJ, the protein coded by mec right-extremity junction (MREJ) (containing the right-extremity of SCCmec and orfX, chromosomal *S. aureus* gene).

**Table 6:** Comparison between BCID2 panel and VITEK2 system in detecting antimicrobial resistance genes.

Target Gene	Target Organism	NPA (95% CI)	PPA (95%CI)
*bla*OXA-48			
	*E. coli*	99% (94–100)	100% (16.7 100)
	*K. pneumoniae*	99% (93.6–100)	100% (67.9–100)
	*Acinetobacter* spp.	100% (95.7–100)	ND
*bla*NDM			
	*E. coli*	99% (94–100)	100% (16.7–100)
	*K. pneumoniae*	100% (95.2–100)	100% (69.9–100)
	*Acinetobacter* spp.	100% (95.6–100)	100% (16.7–100)
*bla*CTX-M			
	*E. coli*	100% (95.2–100)	75% (46.1–91.7)
	*K. pneumoniae*	99% (93.3–100)	86.6(60.8–97.5)
*mecA/C*			
	*S. aureus*	100% (95.7–100)	ND
	*Staphylococcus* spp.	100% (94.1–100)	100% (83–100)
*MecA/C and MERJ*			
	*S. aureus*	100% (95.5–100)	100% (45–100%)
	*Staphylococcus* spp.	100% (95.7–100)	ND
Total		99.6% (99–100)	90% (81.4–95)

*blaCTX-M*, *extended spectrum beta-lactamase (cefotaximase*); *bla*NDM, New Delhi metallo-β-lactamase; *bla*OXA-48, oxacillinase type carbapenemase; *mec*A/*mec*C, a gene A or C that produces a mutated pencillin binding protein coded for methicillin resistance; MREJ, the protein coded by *mec* right-extremity junction (MREJ) (containing the right-extremity of SCCmec and orfX, chromosomal *S. aureus* gene); ND, not Detected.

## Data Availability

All the data supporting the findings are included in the manuscript.
